# BMSC-Derived Exosomes Ameliorate LPS-Induced Acute Lung Injury by miR-384-5p-Controlled Alveolar Macrophage Autophagy

**DOI:** 10.1155/2021/9973457

**Published:** 2021-06-13

**Authors:** Xuan Liu, Chengjin Gao, Yang Wang, Lei Niu, Shaowei Jiang, Shuming Pan

**Affiliations:** ^1^Department of Nephrology, Shanghai General Hospital, Shanghai Jiao Tong University School of Medicine, No. 100 Haining Road, Shanghai 200080, China; ^2^Department of Emergency, Xinhua Hospital, Shanghai Jiao Tong University School of Medicine, No. 1665 Kongjiang Road, Shanghai 200092, China

## Abstract

Acute lung injury (ALI) and acute respiratory distress syndrome (ARDS) are common critical diseases. Bone marrow mesenchymal stem cell (BMSC) transplantation is previously shown to effectively rescue injured lung tissues. The therapeutic mechanism of BMSC-derived exosomes is not fully understood. Here, we investigated the BMSC-derived exosomal microRNAs (miRNAs) on effecting lipopolysaccharide- (LPS-) induced ALI and its mechanism. *In vitro*, rat alveolar macrophages were treated with or without exosomes in the presence of 10 *μ*g/ml LPS for 24 h. Cell viability was determined with Cell Counting Kit-8 assay. Apoptotic ratio was determined with TUNEL and Annexin V-FITC/PI double staining. The levels of miR-384-5p and autophagy-associated genes were measured by RT-qPCR and western blot. Autophagy was observed by TEM and assessed by means of the mRFP-GFP-LC3 adenovirus transfection assay. *In vivo*, we constructed LPS-induced ALI rat models. Exosomes were injected into rats via the caudal vein or trachea 4 h later after LPS treatment. The lung histological pathology was determined by H&E staining. Pulmonary vascular permeability was assessed by wet-to-dry weight ratio and Evans blue dye leakage assay, and inflammatory cytokines in serum and BALF were measured by ELISA. Furthermore, the therapeutic mechanism involved in miR-384-5p and Beclin-1 was determined. The results showed that BMSC-derived exosomes were taken up by the alveolar macrophages and attenuated LPS-induced alveolar macrophage viability loss and apoptosis. Exosomes effectively improved the survival rate of ALI rats within 7 days, which was associated with alleviating lung pathological changes and pulmonary vascular permeability and attenuating inflammatory response. Furthermore, this study for the first time found that miR-384-5p was enriched in BMSC-derived exosomes, and exosomal miR-384-5p resulted in relieving LPS-injured autophagy disorder in alveolar macrophages by targeting Beclin-1. Therefore, exosomal miR-384-5p could be demonstrated as a promising therapeutic strategy for ALI/ARDS.

## 1. Introduction

Acute lung injury (ALI) is a common critical disease that may develop into highly lethal acute respiratory distress syndrome (ARDS), with progressive respiratory failure and intractable hypoxemia as clinical characteristics [1]. The cellular pathology of ALI mainly involves the destruction of alveolar-capillary membrane barrier, a mass of neutrophil migration, and imbalances of proinflammatory and anti-inflammatory cytokines in the lungs [2, 3]. Alveolar macrophages are described as a major component of airspace leukocytes and critically influence the development of ALI following infection and noninfectious stimuli [4]. Current clinical therapy for ALI includes pathogenesis removal, inflammation regulation, lung-protective ventilation, and organ supportive treatment as soon as possible to prevent the lung tissue injury [5, 6]. Despite tremendous efforts and research, there is no real progress of improving ALI prognosis [7]. Thus, searching for new and effective methods for the treatment of ALI/ARDS has become an urgent subject to be studied.

Numerous studies provide evidence to demonstrate a therapeutic effect of bone marrow mesenchymal stem cells (BMSCs) in various clinical disorders, including ALI, in which BMSCs prevent tissue injury through different mechanisms [8, 9]. Recently, several researches have indicated that the BMSCs secrete specialized membranous nanosized vesicle termed exosomes to prevent lung tissue injury [10–12]. Exosomes are small (30-100 nm in diameter) membrane-bound vesicles, merging their membrane contents into the recipient cell membrane and delivering diverse biomolecules, including lipids, proteins, and nucleic acids [13]. The exosomal surface molecules and contents vary from different physiological and pathological processes; the difference may lead to completely reversed fate of target cells [14, 15]. Although BMSC-derived exosomes have been considered as promising therapeutic tools of ALI, the mechanism by which exosomes improved the prognosis of ALI is not entirely understood.

Among the contents of exosomes, microRNAs (miRNAs) are considered as small endogenous noncoding RNAs that regulate gene expression posttranscriptionally through specifically identifying the 3′-untranslated region (3′-UTR) of the mRNA, and play a crucial role in modulation of physiological and pathological processes [16–18]. Herein, we hypothesized that the protective effects of BMSC-derived exosomes in ALI are partially through the transfer of miRNA from the exosomes to the target cells. We were particularly interested in miR-384-5p because of previous work from our group as well as other investigators who have reported that miR-384-5p is a newly recognized one that may participate in mediating cell autophagy in various diseases [19–21].

Autophagy is a highly conserved pathway to degrade and recycle intracellular components for controlling eukaryotic cell metabolism and survival in physiological processes [22]. In addition, autophagy exerts a housekeeping role in maintaining cellular homeostasis, where it is defined as an intracellular quality-control supervisor to remove long-lived or misfolded proteins and damaged organelles. There is a complex interrelation between autophagy modulation and cellular energy balance [23, 24]. In the last few years, accumulating evidence has demonstrated that the stabilization of autophagy appears to be protective against multiple tissue damage. However, whether BMSC-derived exosomes could regulate autophagy of target cells in ALI is unclear.

Herein, the present research was performed with the aim of exploring whether BMSC-derived exosomes had preventative effects against lipopolysaccharide- (LPS-) induced ALI and the underlying mechanism using both *in vitro* and *in vivo* assays. Our findings indicated that BMSCs secreted exosomes to transfer miR-384-5p into alveolar macrophages, which further protected against ALI by alleviating autophagy stress of alveolar macrophages via downregulation of Beclin-1.

## 2. Materials and Methods

### 2.1. Ethics Statement

Adult male Sprague-Dawley rats (6-8 weeks old, weighing 220 ± 20 g) were purchased from the Animal Centre of Chinese Academy of Sciences (Shanghai, China) and raised in specific pathogen-free animal cages under constant temperature and humidity and a 12 h/12 h dark/light cycle with adequate food and water. All experimental protocols with regard to the use of animals were approved by the Institutional Animal Care Committee of Shanghai Xinhua Hospital. All animal experiments were performed in accordance with the guidelines of decreasing the amount of suffering, pain, and discomfort of the experimental animals.

### 2.2. Isolation, Characterization, and Differentiation of BMSCs

The bone marrow from SD rats was flushed with DMEM/F12 and then isolated by centrifugation at 800 × g for 5 min. Subsequently, the sediments were harvested and seeded at 1 × 10^5^ cells/cm^2^ in DMEM/F12 with 10% heat-inactivated fetal bovine serum (FBS; Sigma-Aldrich, St Louis, MO, USA) plus 100 U/ml penicillin and 100 *μ*g/ml streptomycin (Invitrogen, Carlsbad, CA, USA) under a humidified atmosphere at 37°C and 5% CO_2_. Medium was changed every 3-4 days after initial plating. When confluence reached approximately 80-90%, the cells were passaged for further expansion.

For detecting the classical biomarkers of BMSCs, we performed flow cytometric analysis by the following fluorescein isothiocyanate- (FITC-) conjugated or phycoerythrin- (PE-) conjugated antibodies: CD29, CD44, CD90, CD105, CD11b/c, CD34, and CD45 (Becton Dickinson, San Jose, CA, USA). The FITC-IgG and PE-IgG isotypic immunoglobulins were detected as isotype controls. After incubation, cells were washed twice and finally suspended in FACS buffer for flow cytometry analysis (BD Biosciences).

The multipotent differentiation potential of BMSCs to differentiate to osteoblasts, adipocytes, and chondroblasts was evaluated. After subculturing to the third generation, the culture medium was replaced with osteogenic, adipogenic, or chondrogenic differentiation complete medium (Cyagen Biosciences, China). After the induction of the differentiation cultures for 14 days, the accumulation of calcium, intracellular lipids, and mucopolysaccharides was estimated by the alizarin red staining, oil red O staining, and Alcian blue staining (Sigma-Aldrich, USA), respectively.

### 2.3. Exosome Extraction and Identification

The exosomes derived from BMSCs were isolated with the exosome isolation kit (Invitrogen, USA). In brief, culture media supplemented with exosome-depleted FBS were used for cultivating BMSCs. Subsequently, conditioned supernatants from BMSC cultures were collected and exosomes were isolated by centrifugation at 3000 rpm for 15 min, followed by filtration with 0.22 *μ*m filter paper. Exosomes were precipitated through Exo Quick TC (System Biosciences) according to the manufacturer's recommendation; then exosomes were resuspended on the basis of the final counts of BMSCs (10 *μ*l per 3 ×10^6^ cells) and stored at -80°C prior to further analysis. The total protein contents of the exosomes were evaluated.

Analysis of particle size and intensity was conducted with nanoparticle trafficking analysis (NTA) system (NanoSight NS300). After isolation, the exosomes were diluted in filtered PBS before administration. Samples were administered and recorded under controlled flow by the NanoSight syringe pump. Automatic settings were performed to measure the minimum particle size and track length. The measuring conditions were temperature 23.75 ± 0.5°C, 25 frames per second, and measuring time 60 s. The detection threshold was uniform in the different groups.

For morphologic observation with transmission electron microscopy (TEM), exosome pellets were seeded on formvar carbon-coated 200-mesh copper electron microscopy grids, placed at room temperature for 5 min, and then were stained with aqueous uranyl acetate. The grids were washed with PBS and continued to semidry at room temperature prior to detection under TEM (Hitachi, H7500 TEM, Tokyo, Japan).

The Alix, CD63, and CD9 biomarkers, constantly anchored on the surface of exosomes, were measured with western blotting analysis. Exosomes were collected and resolved by SDS/PAGE and then transferred to PVDF membranes (Millipore, Billerica, MA, USA). The membranes were blocked by 5% nonfat milk in TBST buffer and incubated overnight using rabbit anti-Alix (1 : 1000, Abcam, Cambridge, MA, USA), CD63 (1 : 500, Santa Cruz Biotechnology, Dallas, TX, USA), and CD9 (1 : 1000, Abcam, Cambridge, MA, USA) separately, then washed with TBST, and incubated continuously using HRP linked goat anti-rabbit IgG (1 : 5000, Cell Signaling Technology, Danvers, MA, USA), and the protein intensity was determined with the automatic imager (General Electric, Fairfield, CT, USA).

### 2.4. Exosome Label and Track

To determine whether BMSC derived-exosomes can be taken up by alveolar macrophages, BMSC derived-exosomes were labeled with Exosome Labeling Kits (System Biosciences, CA, USA), which allow us to fluorescently label isolated exosomes to track cellular interaction and uptake. Exosome Labeling Kits consist of Exo-red and Exo-green. The Exo-red, which labels nucleic acids and produces a red fluorescence, is based on acridine orange chemistry. The Exo-green, which labels proteins within exosomes and fluorescent green, is based on carboxyfluorescein succinimidyl diacetate ester (CFSE) chemistry. The procedure in our study was following the manufacturer's instructions.

### 2.5. Cell Culture and Study Groups *In Vitro*

Rat alveolar macrophage line NR8383 was purchased from the Cell Resource Center of Shanghai Institutes for Biological Sciences, Chinese Academy of Sciences (Shanghai, China). Alveolar macrophages were planted in 25 cm^2^ polystyrene flasks containing with DMEM/F12 medium, 10% heat-inactivated FBS, 100 U/ml penicillin, and 100 *μ*g/ml streptomycin under a humidified atmosphere of 95% air and 5% CO_2_. Culture medium was replaced every 48 h, and culture was split at a ratio of 1 : 4 once a week.

Subsequently, the cells were exposed to increasing concentrations (0.01-100 *μ*g/ml) of LPS for 24 h. Cell Counting Kit-8 assay was subsequently performed; then, 10 *μ*g/ml LPS was selected for the further mechanistic study. Four experimental groups were continually treated for 24 h as follows: (1) control: alveolar macrophages were incubated under normal condition; (2) LPS: cells were treated with 10 *μ*g/ml LPS to establish a cell-injury model; (3) LPS+non-Ex: cells were treated with exosome-depleted BMSC-conditioned media (20 *μ*l/ml) in the presence of 10 *μ*g/ml LPS; and (4) LPS+Ex: cells were cocultured with BMSC-derived exosomes (20 *μ*l/ml) in the presence of 10 *μ*g/ml LPS.

### 2.6. Determination of Cell Viability

Cell viability was quantitatively evaluated by Cell Counting Kit-8 (Dojindo, Tokyo, Japan). In brief, alveolar macrophages (1 × 10^4^) were planted in 96-well microculture plates that had been precoated with poly-L-lysine and incubated overnight. Then, the viability assay was conducted, and absorbance was assessed using a microplate reader at a wavelength of 450 nm according to the manufacturer's instructions with some modification. Three independent experiments were performed, and cell viability of different groups was evaluated as a percentage of the control.

### 2.7. Detection of Apoptotic Ratio

To evaluate the LPS-induced cell apoptosis, in situ TUNEL staining assay was performed using a commercially available kit. For nuclear counterstaining, DAPI was used in terms of the manufacturer's instructions. The fluorescence was detected with a Leica DMI-3000B phase-contrast fluorescence microscope.

For quantitative analysis, apoptotic ratios were evaluated by the Annexin V-FITC/PI Apoptosis Detection Kit according to the manufacturer's recommendations. FITC and PI fluorescence was detected by a flow cytometer (BD Bioscience) with an excitation wavelength of 488 nm and an emission wavelength of 530 nm.

### 2.8. Assessment of Autophagy

Autophagic vacuoles in alveolar macrophages were observed by a transmission electron microscope (TEM). The cells were fixed by 2.5% glutaraldehyde and cut into 1 mm × 1 mm × 1 mm pieces. Subsequently, the cells were dehydrated, embedded, sliced, stained, and then observed under the TEM (Hitachi H-7650, Japan). The following detection was conducted by pathologists who were blinded to the groups. The typical autophagic vacuoles of each cell sample were observed.

For quantitative analysis, autophagy flux was detected using means of the mRFP-GFP-LC3 adenovirus transfection analysis. Alveolar macrophages were transduced for 48 h with an adenovirus expressing mRFP-GFP-LC3 (tfLC3) (HanBio, Shanghai, China) with a MOI of 100 and then treated with other agents or left untreated. The cells were observed using a fluorescence microscope. The amounts of autophagosome and autolysosome dots were assayed via counting 50 cells in a minimum of three experiments.

### 2.9. Immunofluorescent Staining

Alveolar macrophages were fixed with 4% paraformaldehyde and then incubated with 0.5% Triton X-100 and 3% Bovine Serum Albumin. Next, cells were incubated with diluted primary antibodies including rabbit anti-Beclin-1 (1 : 1000, Cell Signaling Technology) overnight at 4°C. After being washed three times in PBS for 5 min each, the cells were incubated with Donkey anti-Rabbit IgG (H+L) Secondary Antibody (Alexa Fluor 488). The fluorescence was determined under a Leica DMI-3000B phase-contrast fluorescence microscope.

### 2.10. Quantitative Real-Time Polymerase Chain Reaction (RT-qPCR)

Alveolar macrophages were collected and treated with total RNA isolation by Trizol Reagent, followed by reverse transcription to cDNA. cDNA was synthesized by Prime Script RT Master Mix (Takara Biotechnology, Dalian, China). Quantitative PCR was conducted to detect the mRNA levels of Beclin-1, Atg7, Atg5, LC3B, and P62 using SYBR Green Ex Taq™ (Takara). GAPDH was used for the normalization of mRNA expression. All of the gene sequences were obtained through GenBank, and primers were devised (Primer premier 6.0) and synthesized (Invitrogen). The forward (F) and reverse (R) primers were as follows: Beclin-1, 5′- GCCTCTGAAACTGGACACG-3′ (F), 5′-CCTCTTCCTCCTGGC TCTCT-3′ (R); Atg7, 5′-ACTGTGCTGGTCTCCTTGCT-3′ (F), 5′-CAGGGTG CTGGGTTAGGTTA-3′ (R); Atg5, 5′-CTCTGCCTTGGAACATCACA-3′ (F), 5′-AGCGTCAGCTTCCTTCACAC-3′ (R); LC3B, 5′-AGAGCGATACAAGGG TGAGAA-3′ (F), 5′-CACTTCAGAGATGGGTGTGG-3′ (R); P62, 5′-CTGTGGT GGGAACTCGCTAT-3′ (F), 5′-AAGGGGTTGGGAAAGATGAG-3′ (R); GAPDH, 5′-ATGACTCTACCCACGGCAAG-3′ (F), 5′-TACTCAGCACCAGCATCACC-3′ (R).

In addition, miRNAs were extracted with mirVana miRNA isolation kit (Ambion, Carlsbad, CA, USA), reverse transcribed through specific RNA-tailing primers (Applied Biosystems, Carlsbad, CA, USA), and quantified with real-time PCR using the TaqMan MicroRNA assay kit (Applied Biosystems). U6 was used for the normalization of miRNA expression. The primers were as follows: miR-384-5p, 5′-CGTGTAAACAATTCCTAGGCAATGT-3′.

### 2.11. Western Blot Analysis

The proteins were harvested and lysed in RIPA buffer. Equal amounts of protein extracts (30 *μ*g) were loaded per lane and resolved by SDS/PAGE. Subsequently, polypeptides were separated and transferred to PVDF membranes. The membranes were blocked and then treated overnight with rabbit anti-Beclin-1 (1 : 1000, Cell Signaling Technology), rabbit anti-Atg7 (1 : 1000, Cell Signaling Technology), rabbit anti-Atg5 (1 : 1000, Cell Signaling Technology), rabbit anti-P62 (1 : 1000, Cell Signaling Technology), rabbit anti-LC3II/I (1 : 1000, Sigma), and mouse anti-*β*-actin (1 : 1000, Beyotime Institute of Biochemistry) antibodies, respectively. After being washed with TBST three times, the membranes were incubated using the corresponding HRP-conjugated secondary antibodies (1 : 1000, Beyotime Institute of Biochemistry) with blocking solution at room temperature for 2 h. Finally, the expression levels of protein were measured by enhanced chemiluminescence kit (Millipore). The protein bands were normalized by *β*-actin and quantified as the ratio of the optical density.

### 2.12. ALI Rat Model and Study Groups *In Vivo*

Adult male Sprague-Dawley rats (6-8 weeks old, body weight 220 ± 20 g) were used in the experiments. After administration of anesthesia by sodium pentobarbital, the rats underwent intratracheal instillation of a nonlethal dose of LPS from E. coli O111:B4 (Sigma-Aldrich, St. Louis, MO, USA) at 5 mg/kg dissolved in 100 *μ*l PBS to induce an ALI model. We chose an initial dose of 50 *μ*l exosomes, which are the amounts of exosomes released by 1.5 × 10^7^ BMSCs to maximize the therapeutic potential.

The treatment groups of ten rats each, which were given simultaneously, were as follows: (1) sham: 100 *μ*l PBS was intratracheally injected; (2) sham+Ex (V): 4 h later after PBS infusion, 50 *μ*l exosomes were intravenously injected through the caudal vein; (3) sham+Ex (T): 4 h later after PBS infusion, 50 *μ*l exosomes were intratracheally injected; (4) LPS: LPS at 5 mg/kg dissolved in 100 *μ*l PBS was intratracheally injected to induce ALI; (5) LPS+Ex (V): 4 h later after LPS treatment, 50 *μ*l exosomes were intravenously injected; and (6) LPS+Ex (T): 4 h later after LPS treatment, 50 *μ*l exosomes were intratracheally injected.

### 2.13. Evaluation of Lung Histological Pathology

To assess the severity of lung injury, the lung tissues were collected at 24 h after treatment. The lung lobes were fixed with 4% paraformaldehyde and embedded in paraffin and then cut into 4 *μ*m sections. Then, the sections were deparaffinized and stained with hematoxylin and eosin (H&E). The severity of lung injury was evaluated using lung injury score as previously described.

### 2.14. Measurement of Pulmonary Vascular Permeability

The trachea and esophagus were isolated through blunt dissection, and the whole wet lung tissues were obtained and estimated. Then, the tissues were treated at 60°C for 48 h to eliminate the moisture; then, the dry weight was determined, and the lung wet-to-dry weight (*W*/*D*) ratio was measured to assess the pulmonary vascular permeability.

In addition, Evans blue dye leakage assay was also used. Evans blue dye (Sigma-Aldrich, St. Louis, MO, USA) in 1 ml saline was injected into rats via the tail vein at 24 h after exosome infusion. 30 min later, heparinized saline was injected to the right ventricle of the heart for eliminating the dye in the lung vascular system and then, the lung tissues were separated. The tissue was treated with formamide (Sigma Aldrich, St. Louis, MO, USA) at 60°C for 24 h. Finally, the concentration of Evans blue dye in the lung tissue was determined with a spectrophotometer at 630 nm.

### 2.15. Measurement of Inflammatory Cytokines

Serum and BALF levels of tumor necrosis factor-*α* (TNF-*α*), interleukin-1*β* (IL-1*β*), interleukin-6 (IL-6), and interleukin-10 (IL-10) were detected with ELISA kits in accordance with the manufacturer's instructions (Nanjing KeyGen Biotech. Co., Ltd.). Finally, the absorbance was read at 450 nm.

### 2.16. Immunohistochemistry Assay

The lung lobes were embedded in paraffin and cut into 4 *μ*m sections. The sections were incubated with rabbit anti-rat Beclin-1 primary antibody (1 : 200, Santa Cruz, CA, USA) at room temperature for 1 h, followed by hydrogen peroxide treatment for 20 min to remove the activity of endogenous peroxidase. Subsequently, the sections were washed with PBS and then treated using biotinylated goat anti-rabbit IgG for 1 h. After washing with PBS, 3,3-N-diaminobenzidine tertrahydrochloride (DAB, SignalStain) was used for coloration.

### 2.17. Bioinformatics Analysis

Rat Beclin-1 3′-UTR sequences were obtained from the Entrez Nucleotide database (http://www.ncbi.nlm.nih.gov/nuccore). The potential miRNA binding sites in the Beclin-1 3′-UTR were analyzed with the common database including TargetScan (http://www.targetscan.org) and miRanda (http://www.micorrna.org).

### 2.18. Macrophage Transfection of miRNA

Cells were planted in a culture dish and transfected at 70-80% confluence using miR-384-5p mimic (50 nM), miR-384-5p inhibitor (100 nM), or the corresponding negative control through Lipofectamine 2000 reagent (Invitrogen, Carlsbad, CA, USA). The miR-384-5p mimic, inhibitor, and corresponding negative control were devised and purchased from GenePharma (Shanghai, China). Transfection efficiency was evaluated by GFP-labeled oligo control. The expression levels of miRNA were measured with RT-qPCR following transfection.

### 2.19. Luciferase Reporter Assay

Luciferase vectors including the 3′-UTR of Beclin-1 containing the Beclin-1-miR-384-5p response elements and the mutant were obtained from GenePharma. Either miR-384-5p mimic, inhibitor, or corresponding negative control was then transfected into the human embryonic kidney (HEK) 293 cells in the presence of either the wild-type or the mutant reporter plasmid. Luciferase activity was determined with the Dual-Light Chemiluminescent Reporter Gene Assay System (Applied Biosystems) and normalized by the *β*-galactosidase activity.

### 2.20. Statistical Analysis

Statistical analysis was performed with the SPSS 19.0 statistical software (SPSS Inc., Chicago, IL, USA). Figures were drawn by GraphPad Prism software (San Diego, USA). All data were presented as mean ± standard deviation (SD). Statistical comparisons between pairs of groups were measured by a two-tailed Student *t*-test. Statistical comparisons between multiple groups were made using Kruskal-Wallis test followed by the Wilcoxon rank sum test with Bonferroni adjustments (for nonnormal distributions) or a one-way analysis of variance followed by Tukey's *post hoc* test (for normal distributions). Survival rate was evaluated by Log-rank (Mantel-Cox) test. The *P* values for significance were set to 0.05 for all tests.

## 3. Results

### 3.1. Characterization and Differentiation of BMSCs

**Figure 1:**
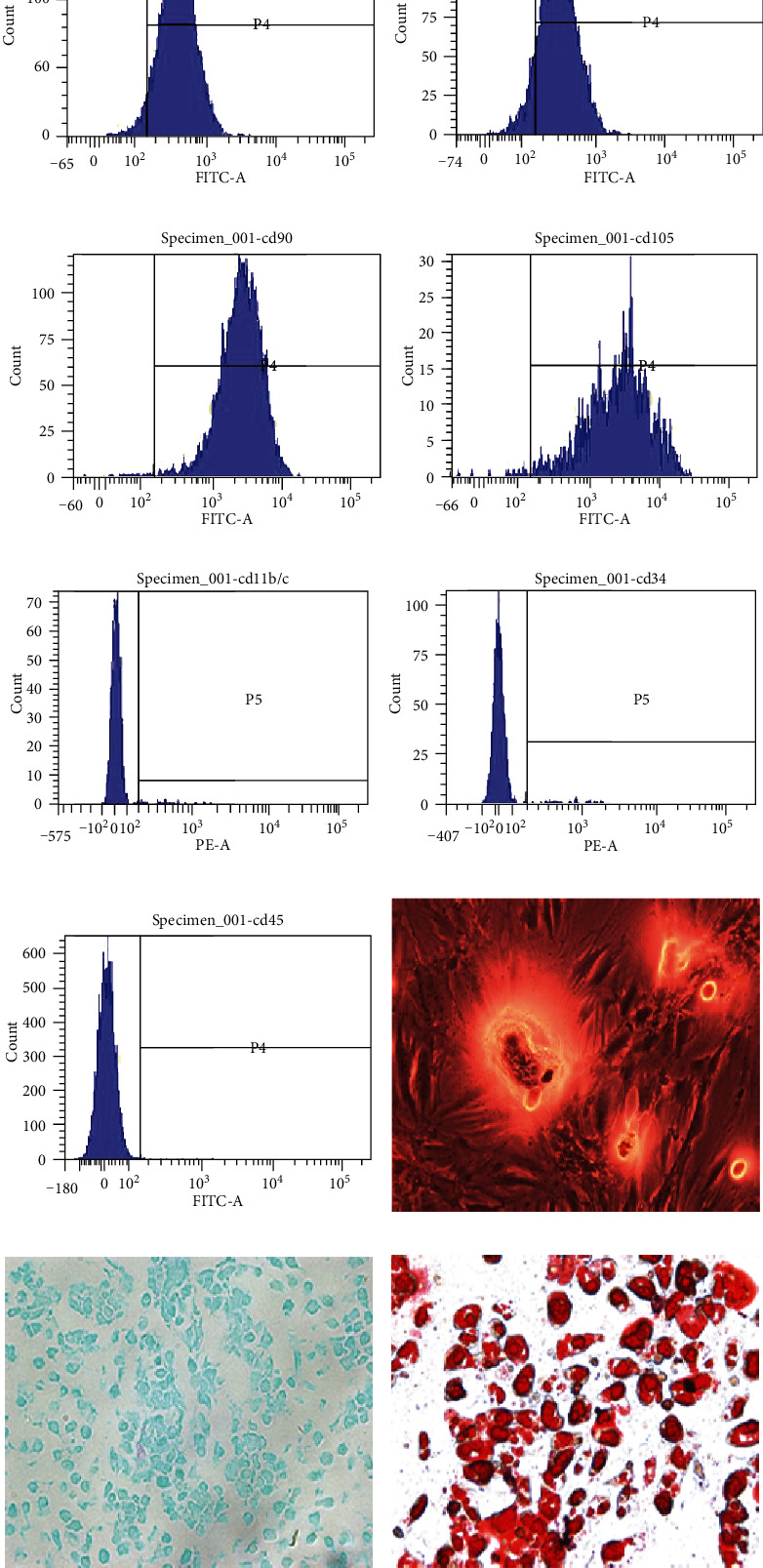
Characterization and differentiation of BMSCs. Fluorescence-activated cell sorting (FACS) analysis demonstrated that BMSCs expressed high levels of CD29 (a), CD44 (b), CD90 (c), and CD105 (d) but were negative for CD11b/c (e), CD34 (f), and CD45 (g). After osteogenic, chondrogenetic, and adipogenic medium induction, the BMSCs showed alizarin red-positive calcium nodule (h), numerous acid mucopolysaccharide (i), or oil-red-O-positive lipid droplets (j), respectively (×200 magnification).

The BMSCs isolated from the rat bone marrow presented as long spindle-shaped fibrocyte-like adherent cells. Fluorescence-activated cell sorting (FACS) analysis illustrated that BMSCs expressed high levels of CD29, CD44, CD90, and CD105 (Figures 1(a)–1(d)) but were negative for CD11b/c, CD34, and CD45 (Figures 1(e)–1(g)). Additionally, after osteogenic, chondrogenetic, and adipogenic medium induction, the BMSCs showed alizarin red-positive calcium nodule (Figure 1(h)), numerous acid mucopolysaccharides (Figure 1(i)), or oil red O-positive lipid droplets (Figure 1(j)), respectively. These results suggest that the BMSCs we obtained are well characterized and identified.

### 3.2. Morphology and Phenotype of BMSC-Derived Exosomes

**Figure 2:**
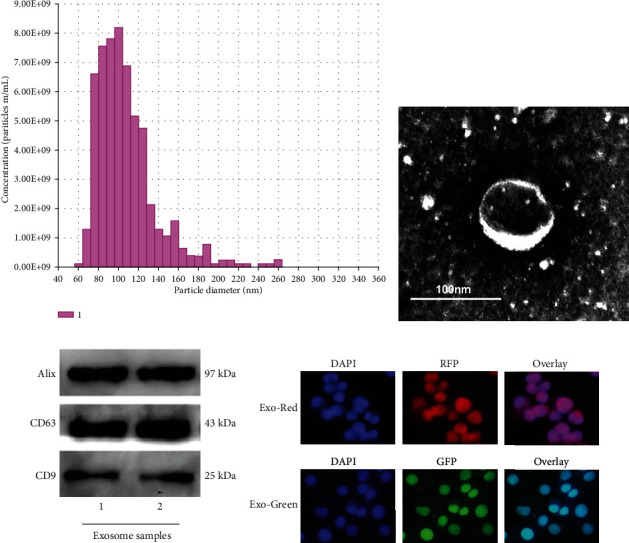
Characterization of BMSC-derived exosomes. (a) Nanoparticle trafficking analysis (NTA) showed the diameters and concentration of exosomes. (b) Transmission electron microscope- (TEM-) analyzed exosomes. The image showed a spheroid shape of approximately 100 nm in diameter. The scale bar represents 100 nm. (c) Western blot-characterized exosomes. BMSC-derived exosome preparation was separated by SDS-polyacrylamide gel electrophoresis, electroblotted to the polyvinylidene fluoride (PVDF) membrane, and probed with exosome markers Alix, CD63, and CD9. (d) Label and track BMSC-derived exosomes. After incubating the labeled Exo-red exosomes with alveolar macrophages for 2 h, the exosome pellet shows strong red fluorescence in the cytoplasm of alveolar macrophages. After incubating the labeled Exo-green exosomes with alveolar macrophages for 24 h, the exosome pellet shows intense green fluorescence in the alveolar macrophages (×400 magnification).

To extract exosomes from BMSCs, the conditioned medium of BMSCs was collected and centrifuged. Then, the morphology and phenotypes of isolated particles were recognized according to the characteristics of exosomes described previously. First, the total number and size distribution of the particles were detected via nanoparticle tracking analysis (NanoSight, Malvern, UK), the results showed that the concentration of the particles was 1.95 × 10^9^ ± 0.21 × 10^9^ particles per ml, and the diameters of the particles were within the range of 63-269 nm, with the average of 108 nm (Figure 2(a)). Secondly, the morphology of the BMSC-derived particles was visualized directly under the transmission electron microscope (TEM); the particles were revealed as round or elliptical nanovesicles with a double-layer membrane structure and diameters about 90 to 100 nm (Figure 2(b)). Finally, the exosome-specific markers of Alix, CD63, and CD9 were evaluated by western blot analysis; all of the three markers were highly expressed in the particles (Figure 2(c)). Therefore, the above properties' analysis showed that BMSC-derived particles isolated in our study were identified as exosomes.

### 3.3. Exosomes Were Taken Up by the Alveolar Macrophages

To investigate whether BMSC-derived exosomes could be taken up by alveolar macrophages, exosomes were labeled with Exosome Labeling Kits (System Biosciences, CA, USA). Exosome Labeling Kits consist of Exo-red and Exo-green. As shown in Figure 2(d), after incubating the labeled Exo-red exosomes with alveolar macrophages for 2 h, the exosome pellet showed strong red fluorescence in the cytoplasm of alveolar macrophages, indicating that a majority of the nucleic acid within exosomes were taken up by the alveolar macrophages. In the similar manner, after incubating the labeled Exo-green exosomes with alveolar macrophages for 24 h, the exosome pellet showed intense green fluorescence in the alveolar macrophages, demonstrating that lots of the protein within exosomes were taken up by the alveolar macrophages. Therefore, the results indicated that BMSC-derived exosomes could be taken up by alveolar macrophages, and the nucleic acids and proteins within exosomes could be delivered to alveolar macrophages.

### 3.4. Exosomes Inhibited LPS-Induced Alveolar Macrophage Viability Loss

**Figure 3:**
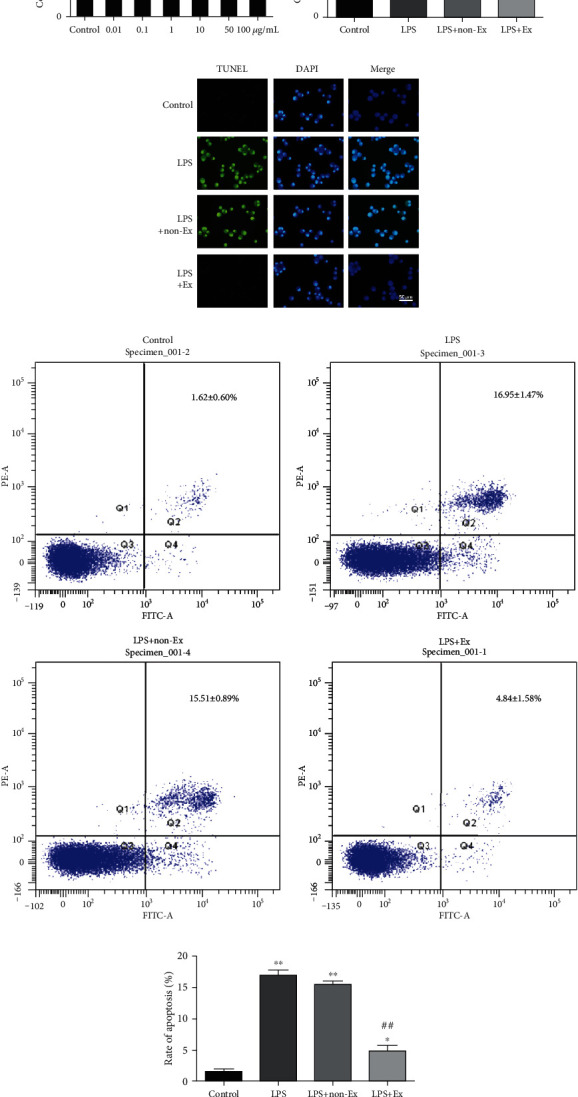
Exosomes inhibited LPS-induced alveolar macrophage viability loss and apoptosis. (a) The optimal action concentrations of LPS on alveolar macrophages were tested by Cell Counting Kit-8 assay. (b) The protective effects of BMSC-derived exosomes on alveolar macrophage viability were estimated by Cell Counting Kit-8 assay. (c) The apoptosis observed by the TUNEL assay in alveolar macrophages. The fluorescent signals from fragmented DNA (green) and nuclei (blue) were visualized and photographed under an inverted fluorescence microscope (×400 magnification). Scale bar represents 50 *μ*m. (d) The apoptosis rate of alveolar macrophages was performed by flow cytometry with Annexin V-FITC/PI double staining. (e) The percentages of apoptotic cells are shown in the bar chart. Values are mean ± SD of three experiments each carried out in triplicate. ^∗^*P* < 0.05 vs. the control group; ^∗∗^*P* < 0.01 vs. the control group; ^#^*P* < 0.05 vs. the LPS group; ^##^*P* < 0.01 vs. the LPS group.

To determine the optimal action concentrations of LPS, alveolar macrophages were exposed to LPS with increasing concentrations from 0.01 to 100 *μ*g/ml for 24 h. As shown in Figure 3(a), the Cell Counting Kit-8 assay revealed that alveolar macrophages treated with 1 *μ*g/ml or lower concentrations of LPS were found to be almost 100% viable, which demonstrated that LPS with varying concentrations from 0.01 to 1 *μ*g/ml did not induce any significant cell viability loss. However, cell viability was significantly decreased after 10 *μ*g/ml LPS treatment in comparison with the control group (53.40% ± 2.06%, *P* < 0.01), indicating that 10 *μ*g/ml LPS is deleterious to alveolar macrophages. 50 or 100 *μ*g/ml LPS decreased cell viability more prominently (44.22% ± 1.71%, 32.55% ± 4.65%, respectively) than 10 *μ*g/ml LPS did, which demonstrated that LPS induced cell viability loss in a dose-dependent manner and that the lowest effective injury dose of 24 h LPS treatment was 10 *μ*g/ml. Thus, 10 *μ*g/ml was selected as the optimal action concentrations of LPS for the further mechanistic study.

Furthermore, we investigated the protective effects of exosomes on alveolar macrophage viability under LPS insult. Our data showed that cell viability decreased prominently after LPS insult (53.77% ± 3.51%, *P* < 0.01 vs. control). Exosome-depleted BMSC-conditioned media (20 *μ*l/ml) did not change the LPS-induced cell viability loss (58.47% ± 3.59%). However, improvements in cell viability were observed (81.99% ± 5.01%) when the 20 *μ*l/ml exosomes were added, compared with the LPS group (*P* < 0.01) (Figure 3(b)). These results indicated that exosomes derived from BMSCs inhibited LPS-induced alveolar macrophage viability loss.

### 3.5. Exosomes Prevented LPS-Induced Alveolar Macrophage Apoptosis

Apoptosis after LPS insult is one of the major pathways that lead to the process of cell death. To provide further evidence that BMSC-derived exosomes may prevent from LPS-induced apoptosis, TUNEL staining was performed. As shown in Figure 3(c), the control group did not exhibit brightly stained condensed nuclei, suggesting no or minimum apoptosis. On the contrary, the proportion of TUNEL-positive nuclei subjected to 10 *μ*g/ml LPS treatment was significantly elevated relative to the control group. Treatment with BMSC-derived exosomes (20 *μ*l/ml) partially recovered the abnormal morphological changes of apoptosis induced by LPS.

Quantitatively, flow cytometry with Annexin V-FITC/PI double staining was performed. As shown in Figures 3(d) and 3(e), the percentage of apoptotic cells in the control group was 1.62% ± 0.60%. However, the apoptotic ratio was as high as 16.95% ± 1.47% after 10 *μ*g/ml LPS insult (*P* < 0.01 vs. control) and markedly decreased to 4.84% ± 1.58% in the LPS+Ex group (*P* < 0.01 vs. LPS), indicating that BMSC-derived exosomes could effectively prevent LPS-induced apoptosis.

### 3.6. Exosomes Relieved LPS-Induced Alveolar Macrophage Autophagy Stress

**Figure 4:**
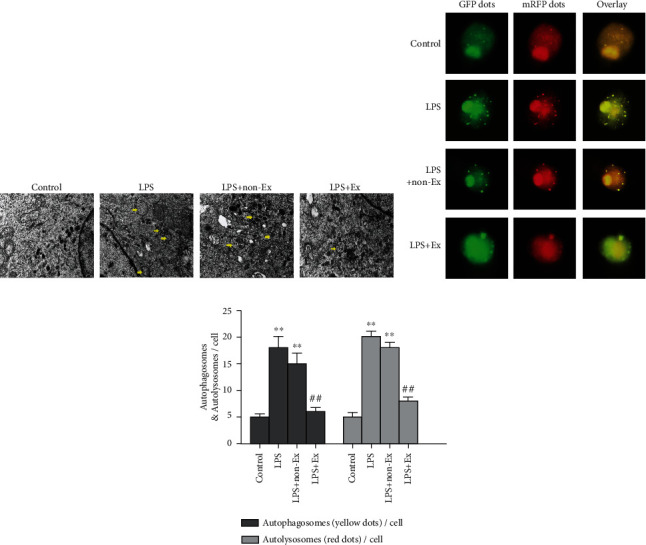
Exosomes relieved LPS-induced autophagy stress of alveolar macrophages. (a) Image of autophagic vacuole in alveolar macrophages taken by a transmission electron microscope (TEM). The yellow arrow indicates typical autophagic vacuoles (×30,000 magnification). (b) Alveolar macrophages were transfected with an adenovirus expressing mRFP-GFP-LC3. Cells were treated with exosomes or exosome-depleted conditioned media and exposed to 10 *μ*g/ml LPS for 24 hours. Yellow puncta (overlay) represent autophagosomes, whereas red puncta indicate autolysosomes (×400 magnification). (c) The numbers of autophagosomes and autolysosomes were determined by counting 50 cells. Values obtained from three independent experiments are expressed as mean ± SD. ^∗^*P* < 0.05 vs. the control group; ^∗∗^*P* < 0.01 vs. the control group; ^#^*P* < 0.05 vs. the LPS group; ^##^*P* < 0.01 vs. the LPS group.

To investigate the effects of BMSC-derived exosomes on autophagy under LPS insults, we used TEM to identify autophagic vacuoles in the alveolar macrophages. The observation revealed that the cellular structure was normal and the mitochondrial membrane integrity was maintained in the control group, while mitochondrial swelling and the double-layer membrane structure of typical autophagic vacuoles were visible in the LPS group and the LPS+non-Ex group. However, treatment with exosomes strongly alleviated LPS-induced autophagy stress as evidenced by a dramatically decreased number of autophagic vacuoles compared with the LPS group (Figure 4(a)).

Furthermore, we measured the autophagy flux of alveolar macrophages by tandem mRFP-GFP-LC3 fluorescence microscopy. Consistently, mRFP-GFP-LC3 punctum formation assays showed that when alveolar macrophages were exposed to 10 *μ*g/ml LPS, autophagosome and autolysosome punctum formations were increased in the LPS group and LPS+non-Ex group, relative to the control group (*P* < 0.01), revealing that LPS enhanced the autophagy flux of alveolar macrophages, and exosome-depleted conditioned media treatment could not change the autophagy. Conversely, treatment with exosomes effectively attenuated autophagosome and autolysosome punctum formation in alveolar macrophages under LPS insults (*P* < 0.01) (Figures 4(b) and 4(c)). Therefore, our results indicated that BMSC-derived exosomes could stabilize autophagy flux of alveolar macrophages exposed to LPS conditions.

### 3.7. Exosomes Increased miR-384-5p and Reduced Beclin-1 in Alveolar Macrophages

**Figure 5:**
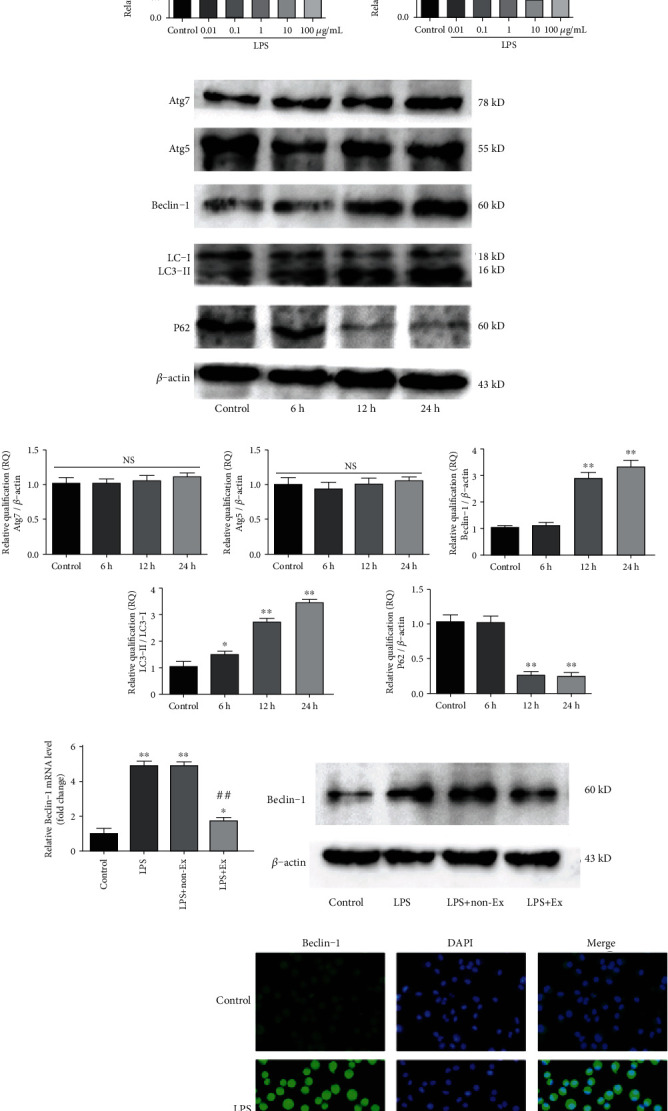
Exosomes increased miR-384-5p and reduced Beclin-1 in alveolar macrophages. (a, b) Protein expressions of Atg7, Atg5, Beclin-1, LC3II/I, P62, and *β*-actin were assessed by western blot in alveolar macrophages treated with gradient concentrations of LPS. Relative quantification (RQ) analyses of the induction in the protein expression were evaluated. (c, d) Protein expressions of Atg7, Atg5, Beclin-1, LC3II/I, P62, and *β*-actin were assessed by western blot in alveolar macrophages treated with LPS for different action times. Relative quantification (RQ) analyses of the induction in the protein expression were evaluated. (e) The mRNA levels of Beclin-1 were detected by RT-qPCR. (f, g) The protein levels of Beclin-1 were detected by western blot. *β*-Actin levels were measured to ensure equal protein loading. (h) Immunofluorescence was used to assess the expression and subcellular localization of Beclin-1 (green fluorescence) using a fluorescence microscopy. DAPI-staining nuclei (blue) were also shown (×400 magnification). Scale bar represents 50 *μ*m. (i) The relative levels of miR-384-5p in BMSC-derived exosomes were detected by RT-qPCR. (j) The relative levels of miR-384-5p in alveolar macrophages were detected by RT-qPCR. Values obtained from three independent experiments are expressed as mean ± SD. ^∗^*P* < 0.05 vs. the control group; ^∗∗^*P* < 0.01 vs. the control group; ^#^*P* < 0.05 vs. the LPS group; ^##^*P* < 0.01 vs. the LPS group.

We examined the protein levels of autophagy-associated genes in alveolar macrophages after LPS insults of different doses (0.01, 0.1, 1, 10, 100 *μ*g/ml) (Figures 5(a) and 5(b)) and action time (6 h, 12 h, 24 h) (Figures 5(c) and 5(d)). Specifically, we analyzed the levels of major autophagy-associated activators Atg7, Atg5, Beclin-1, and LC3-II/I and autophagy-associated degradation protein P62. We found that the Beclin-1 protein levels and LC3-II/I ratio significantly increased following 24 h of 1-100 *μ*g/ml LPS treatment, than those from the control group (*P* < 0.01). Furthermore, the results showed that 1-100 *μ*g/ml LPS stimulated autophagosome-lysosome fusion as evidenced by degradation of P62 after LPS treatment (*P* < 0.01). However, the protein expression levels of Atg7 and Atg5 were unchanged. The mRNA levels of autophagy-associated gene detected by RT-qPCR were in accordance with the results of western blot.

In the following experiments, we explored the effects of BMSC-derived exosomes on the mRNA and protein levels of Beclin-1 in LPS-exposed alveolar macrophages. As shown in Figures 5(e)–5(g), in comparison with the control group, 10 *μ*g/ml LPS promoted mRNA and protein levels of Beclin-1 (*P* < 0.01). However, treatment with 20 *μ*l/ml exosomes effectively reduced LPS-induced Beclin-1 excessive elevation (*P* < 0.01 vs. the LPS group) by RT-qPCR and western blot analysis. These observations were in line with the results of immunofluorescent staining (Figure 5(h)).

Additionally, RT-qPCR analysis demonstrated that LPS could induce the elevation of miR-384-5p levels in BMSC-derived exosomes in a dose-dependent manner (Figure 5(i)). However, LPS caused a significant decrease of miR-384-5p in alveolar macrophages compared with the control group (*P* < 0.05). After the alveolar macrophages were treated with BMSC-derived exosomes, miR-384-5p expression in alveolar macrophages was increased, compared with nonexosome treatment under LPS insults (*P* < 0.01) (Figure 5(j)). Taken together, the exosomes showed effects on increasing miR-384-5p levels and decreasing Beclin-1 expression in the LPS-exposed alveolar macrophages.

### 3.8. Exosomes Improved Survival Rate in ALI Rats

**Figure 6:**
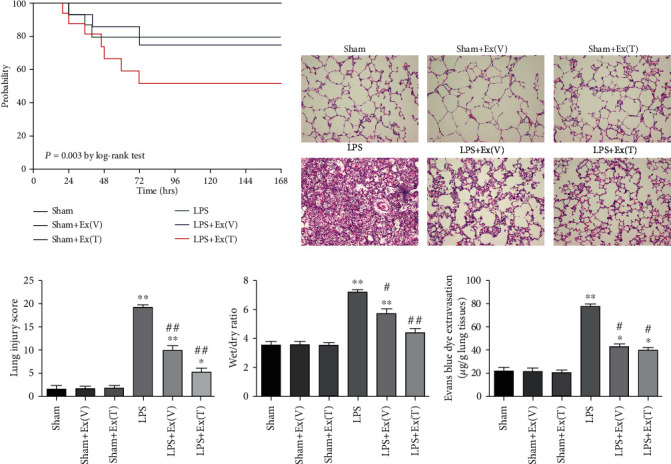
Exosomes alleviated LPS-induced lung injury and attenuated pulmonary vascular permeability in ALI rats. (a) Rat survival rate of 7 days was evaluated by Log-rank (Mantel-Cox) test. (b) H&E staining was used for evaluating the pathological changes in the lung. The image revealed that LPS caused pathological changes in lung tissue, and the administration of the exosomes attenuated lung injury (×100 magnification). (c) The quantitative analysis of the lung injury scores in each group. Lung injury score of LPS-exposed lung turned to be higher than the sham group, while the score was decreased after exosome administration. (d) *W*/*D* ratio in different groups. *W*/*D* ratio of the LPS-exposed lung turned to be higher than the sham group, while the ratio was decreased after exosome administration. (e) The quantitative analysis of Evans blue dye leakage. This result was similar to that of the *W*/*D* ratio, suggesting that exosomes could improve lung vascular permeability impaired by LPS. Values obtained from three independent experiments are expressed as mean ± SD and compared using ANOVA followed by LSD test. ^∗^*P* < 0.05 vs. the sham group; ^∗∗^*P* < 0.01 vs. the sham group; ^#^*P* < 0.05 vs. the LPS group; ^##^*P* < 0.01 vs. the LPS group.

For the survival study, the rats were observed for 7 days. As shown in Figure 6(a), the survival rate in ALI rats remained stable and markedly higher than the rate in normal ones (52.05% vs. 100%, *P* < 0.01). However, the administration of exosomes significantly improved the survival rate in ALI rats within 72 h (*P* < 0.01). Additionally, no significant difference was observed between the LPS+Ex (V) group and LPS+Ex (T) group.

### 3.9. Improved Pathological Changes in Lung Tissue of ALI Rats

H&E staining was used for evaluating the pathological changes in the lung. After LPS stimulation, the image of lung histopathology revealed extensive leukocyte infiltrates in the lung tissue, diffuse interstitial and alveolar edema, remarkable interalveolar septal thickening, lung hemorrhage, hyaline membrane formation in the alveolar space, and alveolar collapse. However, the administration of the exosomes attenuated lung injury as shown by histopathology (Figure 6(b)).

For quantitative analysis, we measured the lung injury score. As shown in Figure 6(c), the score was increased to 19.36 ± 0.95 at 24 hours in the LPS group compared with the sham group (*P* < 0.01). However, the administration of exosomes attenuated lung injury as shown by histopathology as well as the lung injury score (*P* < 0.01). In addition, intratracheal injection of exosomes tended to be more beneficial than intravenous injection on the improvement of the lung injury (*P* < 0.05).

### 3.10. Exosomes Improved Pulmonary Vascular Permeability in ALI Rats

To examine the effects of exosomes on the pulmonary vascular permeability, *W*/*D* ratio was measured. As Figure 6(d) showed, the *W*/*D* ratio in the LPS group was increased dramatically compared with the sham group (*P* < 0.01). After treatment with the exosomes, the *W*/*D* ratio was decreased significantly (*P* < 0.05).

Besides, Evans blue dye extravasation was carried out to assess the large molecular permeability of the lung vascular system. Similarly, LPS stimulation increased Evans blue dye extravasation from lung vascular to lung interstitial and alveolar space (*P* < 0.01). After treatment with the exosomes, Evans blue dye extravasation was decreased statistically (*P* < 0.05) (Figure 6(e)). This result was similar to that of the *W*/*D* ratio, suggesting that exosomes had a beneficial effect on pulmonary vascular permeability in LPS-induced lung injury in rats.

### 3.11. Exosomes Regulated the Inflammatory Cytokines in ALI Rats

**Figure 7:**
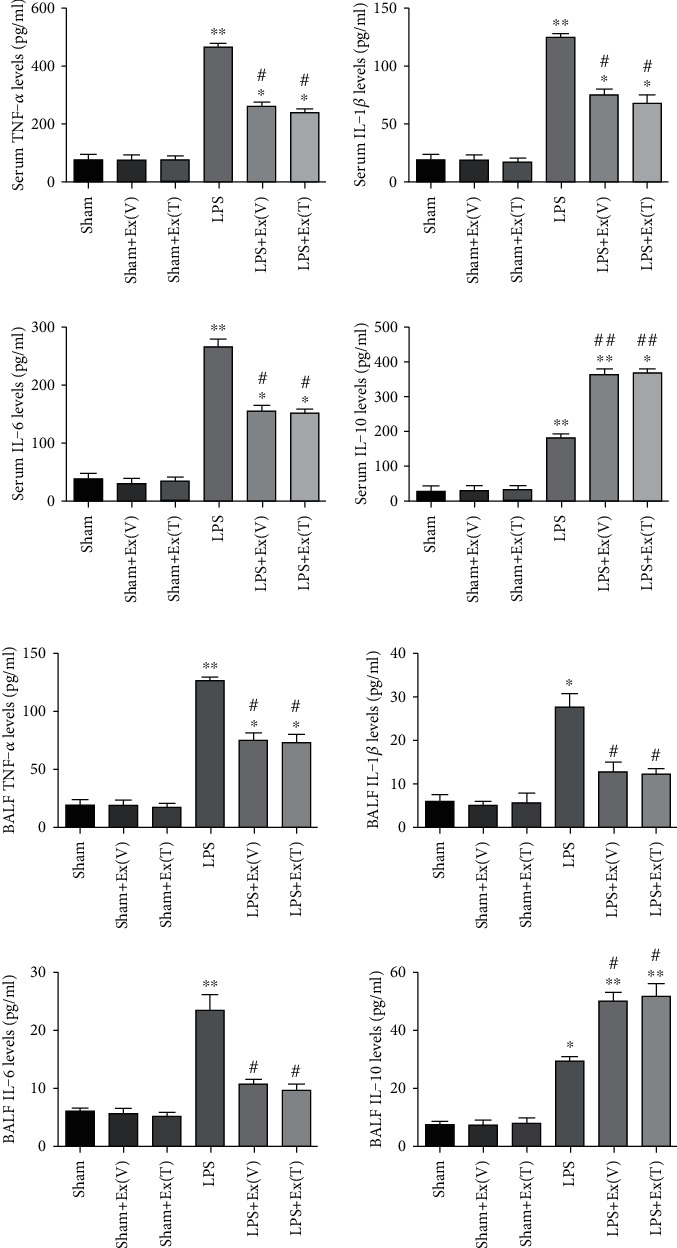
Exosomes regulated LPS-induced inflammatory cytokines in ALI rats. (a) Serum levels of TNF-*α*, IL-1*β*, IL-6 and IL-10 were measured by ELISA. (b) BALF levels of TNF-*α*, IL-1*β*, IL-6 and IL-10 were also measured. The results showed that exosomes attenuated LPS-induced TNF-*α*, IL-1*β* and IL-6 elevation, and promoted IL-10 production in serum and BALF. Values obtained from three independent experiments are expressed as mean ± SD and compared using ANOVA followed by LSD test. ∗*P* < 0.05 vs. sham group; ∗∗*P* < 0.01 vs. sham group; ^#^*P* <0.05 vs. LPS group; ^##^*P* <0.01 vs. LPS group.

To investigate the effects of exosomes on LPS-induced inflammation, inflammatory cytokines in serum and BALF were measured using ELISA. As shown in Figure 7(a), the levels of TNF-*α*, IL-1*β*, IL-6, and IL-10 in serum were dramatically increased after LPS administration compared with the sham group. By contrast, intravenous or intratracheal injection of exosomes reduced TNF-*α*, IL-1*β*, and IL-6 elevation and promoted IL-10 production. No significant difference was observed between the LPS+Ex (V) group and LPS+Ex (T) group. Furthermore, we found that the change trend of inflammatory cytokines in BALF was similar to the change in serum (Figure 7(b)). Therefore, the results indicated that exosomes could effectively attenuate excessive inflammation caused by LPS.

### 3.12. Exosomes Increased miR-384-5p and Reduced Beclin-1 in Lung Tissue of ALI Rats

**Figure 8:**
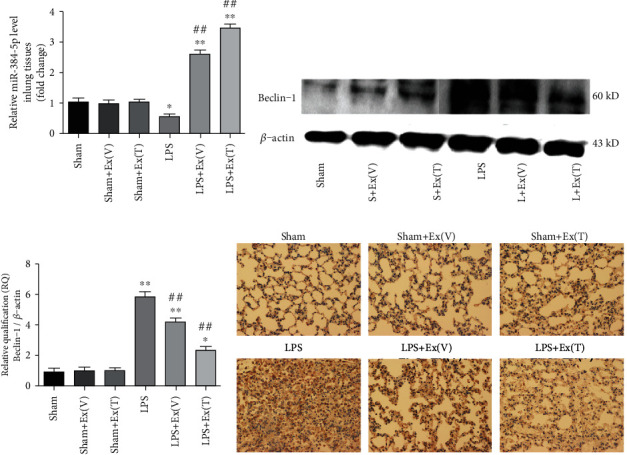
Exosomes increased miR-384-5p and reduced Beclin-1 in lung tissue of ALI rats. (a) The relative levels of miR-384-5p in lung tissues were detected by RT-qPCR. (b, c) The protein levels of Beclin-1 in lung tissues were detected by western blot. *β*-Actin levels were measured to ensure equal protein loading. (d) The protein levels of Beclin-1 in lung tissues were detected by immunohistochemistry (×200 magnification). Values obtained from three independent experiments are expressed as mean ± SD and compared using ANOVA followed by LSD test. ^∗^*P* < 0.05 vs. the sham group; ^∗∗^*P* < 0.01 vs. the sham group; ^#^*P* < 0.05 vs. the LPS group; ^##^*P* < 0.01 vs. the LPS group.

As shown in Figure 8(a), the miR-384-5p levels in rat lung tissue were dramatically decreased after LPS administration compared with the sham group (*P* < 0.05). Nevertheless, intravenous or intratracheal injection of exosomes could significantly elevate the miR-384-5p levels in lung tissue of ALI rats (*P* < 0.01 vs. LPS).

Furthermore, combined with the data from RT-qPCR and western blot assay, we found that the mRNA and protein levels of Beclin-1 in ALI rat lung tissue were obviously higher than those in the sham group (*P* < 0.01). However, intravenous or intratracheal injection of exosomes effectively reclined both mRNA and protein elevations of Beclin-1 in ALI rats (*P* < 0.01 vs. LPS) (Figures 8(b) and 8(c)). Additionally, the results of further immunohistochemistry assay were in accordance with the results of western blot (Figure 8(d)).

### 3.13. miR-384-5p Modulated Beclin-1 Expression

**Figure 9:**
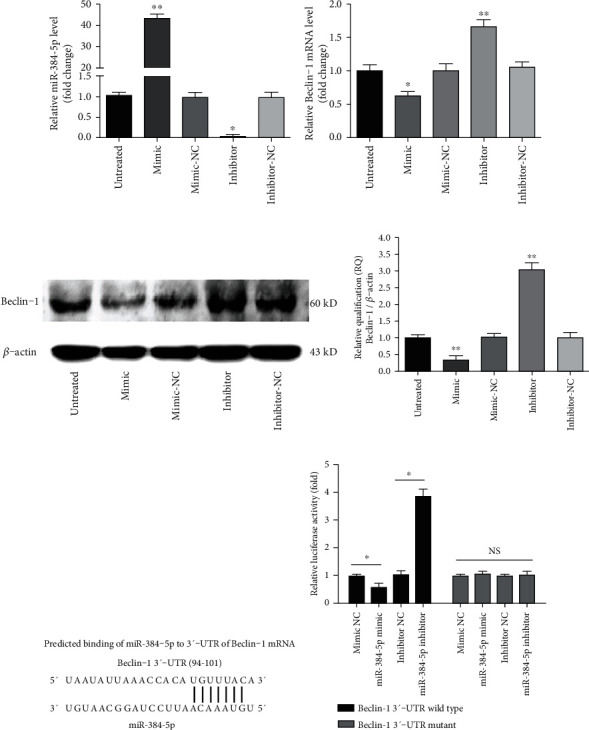
Beclin-1 was one of the target genes of miR-384-5p. (a) The relative levels of miR-384-5p were detected by RT-qPCR after treatment with miR-384-5p mimic, miR-384-5p inhibitor, or the corresponding negative control (NC). (b) The relative mRNA levels of Beclin-1 were detected by RT-qPCR after treatment with miR-384-5p mimic, miR-384-5p inhibitor, or the corresponding NC. (c) The protein levels of Beclin-1 were detected by western blot after treatment with miR-384-5p mimic, miR-384-5p inhibitor, or the corresponding NC. *β*-Actin levels were measured to ensure equal protein loading. (d) Relative quantification (RQ) analyses of the induction in the protein levels of Beclin-1 showed that the miR-384-5p mimic decreased the protein expression level of Beclin-1, whereas the miR-384-5p inhibitor increased the protein level of the protein. (e) Bioinformatics analyses showing that miR-384-5p targets 3′-UTR of Beclin-1 mRNA at one binding site. (f) The relative luciferase value of the interaction between miR-384-5p and the 3′-UTR of Beclin-1. HEK 293 cells were transfected with wild-type or mutant 3′-UTR luciferase constructs and with miR-384-5p mimic, inhibitor, or the corresponding negative control (NC), as indicated (*n* = 6). Values obtained from three independent experiments are expressed as mean ± SD. ^∗^*P* < 0.05 vs. the untreated group; ^∗∗^*P* < 0.01 vs. the untreated group.

To determine whether miR-384-5p can regulate Beclin-1 expression, miR-384-5p mimic, antisense for miR-384-5p (miR-384-5p inhibitor), or the corresponding negative control (NC) was transfected into alveolar macrophages. The miR-384-5p mimic upregulated, whereas the miR-384-5p inhibitor downregulated miR-384-5p levels (Figure 9(a)). RT-qPCR and fluorescence microscopy demonstrated relatively high transfection efficiency (approximately 90%) of the mimic at 50 nM and the inhibitor at 100 nM concentration into alveolar macrophages. By contrast, the oligo negative controls had no effects on miR-384-5p level, either at high or low concentrations.

The miR-384-5p mimic, which upregulated miR-384-5p expression, significantly reduced both the mRNA and protein expression levels of Beclin-1. Nevertheless, the miR-384-5p inhibitor, which decreased miR-384-5p expression, increased both the mRNA and protein expression levels of Beclin-1 (Figures 9(b)–9(d)). Therefore, miR-384-5p may directly regulate Beclin-1 expression at the posttranscriptional level in alveolar macrophages.

### 3.14. miR-384-5p Directly Bound to Beclin-1

Based on several publicly available bioinformatics websites (TargetScan, miRanda and miRBase), Beclin-1 may be the direct target of miR-384-5p. The 3′-UTR of Beclin-1 mRNA have binding sites for miR-384-5p (Figure 9(e)). The minimum free energy values of miR-384-5p and Beclin-1 hybridization via RNAhybrid software were -20.9 kcal/mol. To further determine whether a miR-384-5p binding site in the Beclin-1 3′-UTR mediated the repression, we inserted the Beclin-1 3′-UTR wild-type or a mutated version into a luciferase system. Transfection of the miR-384-5p mimic totally restrained the luciferase activities of the 3′-UTR wild type of Beclin-1; nevertheless, the construct containing a mutant binding site abrogated the inhibitory effect of the miR-384-5p mimic. In parallel, transfection of the miR-384-5p inhibitor increased the luciferase activity, whereas the mutant Beclin-1 3′-UTR abolished the positive effect of the miR-384-5p inhibitor (Figure 9(f)). In conclusion, these results demonstrated that Beciln-1 was one of the target genes of miR-384-5p.

**Figure 10:**
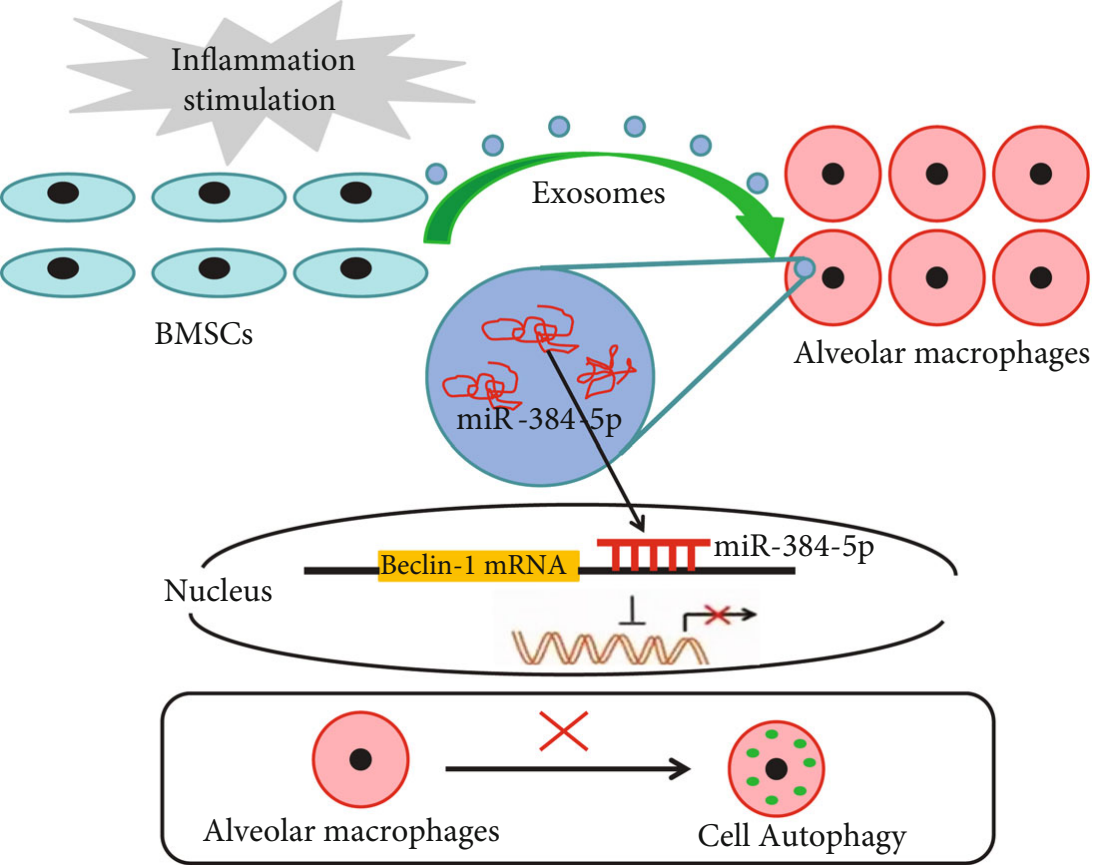
The schematic diagram depicts molecular basis underlying BMSC-derived exosomes on ALI treatment by transferring miR-384-5p. BMSCs can secrete exosomes to transfer miR-384-5p into alveolar macrophages, which further protects against ALI by alleviating autophagy stress of alveolar macrophages via downregulation of Beclin-1.

Therefore, our study suggested that the remission of autophagy stress may result from downregulation of Beclin-1, through increased miR-384-5p that binds and suppresses translation of Beclin-1 mRNA in alveolar macrophages (Figure 10).

## 4. Discussion

ALI and its severe form, known as ARDS, are prevalent and life-threatening diseases caused by direct lung injuries and indirect systemic inflammatory responses arising from a wide variety of situations such as trauma, pneumonia, shock, sepsis, or aspiration of gastric contents [1, 25]. Fundamentally, along with the intricate pathogenesis of ALI, cell necrosis and apoptosis cause the destruction and dysfunction of the barrier properties of the pulmonary endothelium and epithelium, leading to relative high mortality worldwide [2, 3, 26]. Although ALI has been extensively explored for decades, we are still lacking recognized effective pharmacotherapy that can improve the prognosis and raise the life quality of ALI-plagued patients. Emerging evidence has revealed that alveolar macrophages are the dominant innate immune cells in the resolution of inflammation response and tissue repair through the influence on other immune cell populations in the lung [27, 28]. Cell death and tissue inflammation establish a positive feedback cycle, inevitably causing exaggerated inflammatory response and deterioration of tissue damage. It is now clear that alveolar macrophages work in concert in the regulation of lung inflammation [29]. All in all, pharmacological manipulation of alveolar macrophages may potentially serve as a logical therapeutic strategy for ALI/ARDS.

Exosomes are 30 to 100 nm in size and spheroid in shape and are generated by various types of cells [13]. The cell-specific cargoes of exosomes including RNA, proteins, and lipids are considered as the critical signaling molecules and can mediate intercellular communication [13–15]. Most cells can generate exosomes under normal circumstances. However, under pathologic or stressful conditions, the quantity and content of exosomes may be changed [30]. Interest in exosomes has greatly expanded with recent discoveries that miRNAs, which are characterized as a group of short noncoding RNAs, are present and transferred into target cells to modulate cell-to-cell communication and adjust recipient cell's functions [31–33]. Many reports have illustrated that exosomes from BMSCs display a potential role in the treatment of ALI. For instance, a previous study found that BMSC-derived exosomes protected against intestinal ischemia reperfusion-induced ALI via inhibition of the TLR4/NF-*κ*B signaling pathway [10]. Another research indicated that BMSCs transferred exosomal miR-30b-3p to type II alveolar epithelial cells (AECs), which further conferred protective effects against ALI by improving the proliferation and reducing the apoptosis of AECs through downregulation of SAA3 [34]. In addition, with an outbreak of coronavirus-related pneumonia COVID-19 worldwide, it has grown to be a global public-health emergency. ALI/ARDS is the main cause for the COVID-19 infection-related morbidity and mortality [35]. The novel coronavirus or SARS-CoV-2 as named by the International Committee on Taxonomy of Viruses has over 14 million confirmed cases worldwide and has claimed over 600,000 lives [35]. Recently, some clinical trials have been registered to investigate the safety and efficacy of different types of BMSCs and their exosomes for treating ALI/ARDS induced by COVID-19, and these intervention measures are expected to be novel therapeutic approaches for COVID-19 patients through mitigating cytokine storm as well as promoting the regeneration of alveolar tissue, attributed to the intrinsic cytokines and growth factor present in the secretome [35, 36]. These preliminary studies have demonstrated the safety and efficacy of BMSCs and their exosomes in mitigating symptoms associated with COVID-19 [37–39]. Thus, they can be used on compassionate basis, owing to their ability to endogenously repair and decrease the inflammatory reactions involved in the morbidity and mortality of COVID-19. A call for an urgent development on the BMSCs and exosome-based therapeutics specifically targeted towards COVID-19 to ensure the health and survival of human being is strongly recommended. However, whether BMSCs protect lung tissues by transferring exosomal miRNAs and the underlying mechanism is not clearly illustrated. In the present study, we found that exosomes released by BMSCs successfully prevented alveolar macrophage injury and miR-384-5p in BMSC derived-exosomes played a pivotal role in relieving autophagy stress of alveolar macrophages by targeting Beclin-1, leading to protect LPS-induced ALI *in vitro* and *in vivo*.

Autophagy is considered as an ancient and conserved process that degrades and recycles intracellular components or organelles for eukaryotic cell death or survival at diversified insults, whereas its dysfunction often leads to cell damage [22–24]. Previous studies have demonstrated that autophagy flux impairment is one of the mechanisms contributing to the detrimental outcome of autophagy after ALI [40]. The effects of autophagy on the ALI rely on the disease background, phase, or severity of the lung injury, and the balance between inflammatory factors, including proinflammatory cell death and anti-inflammatory factors [41]. Thus, it is imperative to realize that the regulation of autophagy may hold promise for potential therapies for patients with ALI. Recently, accumulating evidence has indicated that miR-384-5p participates in modulating cell autophagy and plays a crucial role in cell survival in response to diverse injury [19–21]. In our study, when the alveolar macrophages were pretreated with BMSC-derived exosomes, the exosomes could be taken up at high efficiency, and the exosomal miR-384-5p was delivered into the alveolar macrophages and participated in regulating the signaling pathways. This delivery successfully caused downstream changes, including the decreased expression of Beclin-1, which is the mammalian orthologue of yeast Atg6 and a core component of the autophagy machinery [42]. Beclin-1 is identified as a landmark protein of autophagy and crucial for the formation and maturation of autophagosomes. Many of these effects are mediated through the activation of specific Beclin-1 binding proteins, including autophagic inducers and autophagic inhibitors [42–45]. Undoubtedly, Beclin-1 dysfunction plays a central role in autophagy disorders. Thus, modulation of Beclin-1 significantly influences the stabilization of autophagy, thereby deeply affecting the cell survival and death [42, 43]. As expected, our study further elucidated that the protective effects of miR-384-5p were connected with suppression of autophagy stress indicated by decreased levels of Beclin-1 in damaged lung tissues and reduced autophagosome formation in alveolar macrophages. Importantly, the abovementioned finding for the first time indicated that BMSC-derived exosomes alleviate LPS-induced ALI by reconstructing the miR-384-5p/Beclin-1 pathway, and exosomal miR-384-5p may be one of the protective factors to prevent alveolar macrophages from autophagy stress. Based on these findings, we speculated that stabilizing the levels of autophagy could provide prospective therapeutic strategies, thus enabling the treatment of an overwhelming autophagy response. In this paper, although the results indicated that exosomal miR-384-5p could play a significant role in the autophagy regulation of recipient cells, we do not rule out altered biological functions of other exosomal cargoes. Since diverse miRNA clusters are involved in ALI, the molecular mechanisms of other exosomal miRNAs in ALI merit further investigation in the future.

## 5. Conclusion

In summary, our data give explanation to the complicated exosome-mediated cross-talk between BMSCs and alveolar macrophages. BMSC-derived exosomes alleviate LPS-induced autophagy stress of alveolar macrophages, at least partly, via delivering exosomal miR-384-5p to alveolar macrophages. Therefore, we get the conclusion that the protective role of exosomal miR-384-5p in the process of ALI is associated with simultaneous suppression of autophagy stress and miR-384-5p could be demonstrated as a potential treatment target for ALI/ARDS.

## Data Availability

The data used to support the findings of this study are included within the article.
